# Polydopamine-coated Au nanorods for targeted fluorescent cell imaging and photothermal therapy

**DOI:** 10.3762/bjnano.10.79

**Published:** 2019-04-01

**Authors:** Boris Nikolayevich Khlebtsov, Andrey Mikhailovich Burov, Timofey Evgenevich Pylaev, Nikolai G Khlebtsov

**Affiliations:** 1 Institute of Biochemistry and Physiology of Plants and Microorganisms, Russian Academy of Sciences, 13 Prospekt Entuziastov, Saratov 410049, Russia; 2 Saratov State University, 83 Ulitsa Astrakhanskaya, Saratov 410026, Russia

**Keywords:** Au nanorods, cancer theranostics, fluorescent bioimaging, folate, polydopamine, targeted phototherapy

## Abstract

Au nanorods (AuNRs) have attracted a great interest as a platform for constructing various composite core/shell nanoparticles for theranostics applications. However, the development of robust methods for coating AuNRs with a biocompatible shell of high loading capacity and with functional groups still remains challenging. Here, we coated AuNRs with a polydopamine (PDA) shell and functionalized AuNR-PDA particles with folic acid and rhodamine 123 (R123) to fabricate AuNR-PDA-R123-folate nanocomposites. To the best of our knowledge, such AuNR-PDA-based composites combining fluorescent imaging and plasmonic phothothermal abilities have not been reported previously. The multifunctional nanoparticles were stable in cell buffer, nontoxic and suitable for targeted fluorescent imaging and photothermal therapy of cancer cells. We demonstrate the enhanced accumulation of folate-functionalized nanoparticles in folate-positive HeLa cells in contrast to the folate-negative HEK 293 cells using fluorescent microscopy. The replacement of folic acid with polyethylene glycol (PEG) leads to a decrease in nanoparticle uptake by both folate-positive and folate-negative cells. We performed NIR light-mediated targeted phototherapy using AuNR-PDA-R123-folate and obtained a remarkable cancer cell killing efficiency in vitro in comparison with only weak-efficient nontargeted PEGylated nanoparticles. Our work illustrates that AuNR-PDA could be a promising nanoplatform for multifunctional tumor theranostics in the future.

## Introduction

Multifunctional imaging and combined multimodal therapy strategies are very promising in cancer theranostics [1–2]. Possible way for such purpose is to integrate various functionalities by incorporating different diagnostic and therapeutic agents into a single core/shell nanoparticle. Au nanorods (AuNRs) have attracted a great interest as a platform for theranostic applications because of tunable optical properties and simple protocols for synthesis with designed parameters [3–4]. The AuNRs themselves can serve as contrast agents for two-photon [5–6], photoacoustic [7–9] and SERS [10–11] imaging, and for plasmonic photothermal therapy (PPT) [12–13]. However, the as-prepared AuNRs demonstrate high toxicity [14–15] and low stability in biological fluids because of a cetyltrimethylammonium bromide (CTAB) bilayer on the AuNR surface, which is a necessary agent in the synthesis method [16]. The coating of the nanoparticles with polymeric or inorganic shells and further functionalization with target molecules can help to overcome this drawback. Meanwhile different imaging and therapeutic agents can be loaded into the shell of multifunctional nanocomposites. Various AuNR-based nanocomposites loaded with anticancer drugs [17–19], photodynamic dyes [20–21], MRI contrast agents [22] and many others ligands [23–24] have already been reported for efficient multimodal cancer treatments both in vitro and in vivo.

An ideal nanorod coating for efficient nanocomposite formation should meet several important criteria. First, the resulting nanoparticles should be nontoxic and colloidally stable in blood serum. Second, the shell should have high loading capacity for various cargo molecules such as drugs or photodynamic dyes. Third, the coating should have functional groups and be ready for click conjugation with target or “shadowing” molecules, e.g., antibodies, peptides, folates and PEG. Finally, the AuNR coating procedure should be robust and provide a tunable shell thickness. The most popular coatings such as mesoporous silica, PEG and polyelectrolyte shells do not meet the above quality criteria.

In 2007 Messersmith et al. reported the mussel-inspired adhesive polydopamine (PDA) multifunctional coating for various materials including nanoparticles [25]. Since that discovery, PDA has received extensive attention owing to its extremely attractive properties. Owing to its simplicity, PDA-assisted coating has been intensively applied for various nanoparticles including nanodiamonds [26], polymeric drug carriers [27], AuNRs [28–34], Fe_3_O_4_ [35], graphene [36], and many others [37–38]. The PDA shell surface contains numerous catechol and quinone groups suitable for click conjugation with various biomolecules through Michael addition and Schiff-base reaction [39–40]. The high loading capacity and biocompatibility of the PDA layer taken together give the opportunity to use AuNR/PDA composites as promising agents for theranostics.

The published examples of fabrication and biomedical applications of PDA-coated nanorods include the following nanoconstructs: (1) AuNR-PDA-Ab for targeted PPT of cells in vitro [28]; AuNR-CuPDA for non-targeted PPT and chemotherapy (via Cu(II) release) [34]; AuNR-PDA-pMBA-Ab for targeted SERS cell imaging [31]; AuNR-PDA-MB/DOX for notargeted combined photodynamic and chemotherapy in vivo [32]; AuNR-PDA-Cisplatin-Iodine125-RGDpeptide for targeted MRI imaging and chemotherapy in vivo [33]. However, AuNR-PDA based nanocomposites that combine fluorescent imaging modality with folate targeting and PPT ability have not been reported in the literature.

**Scheme 1:**
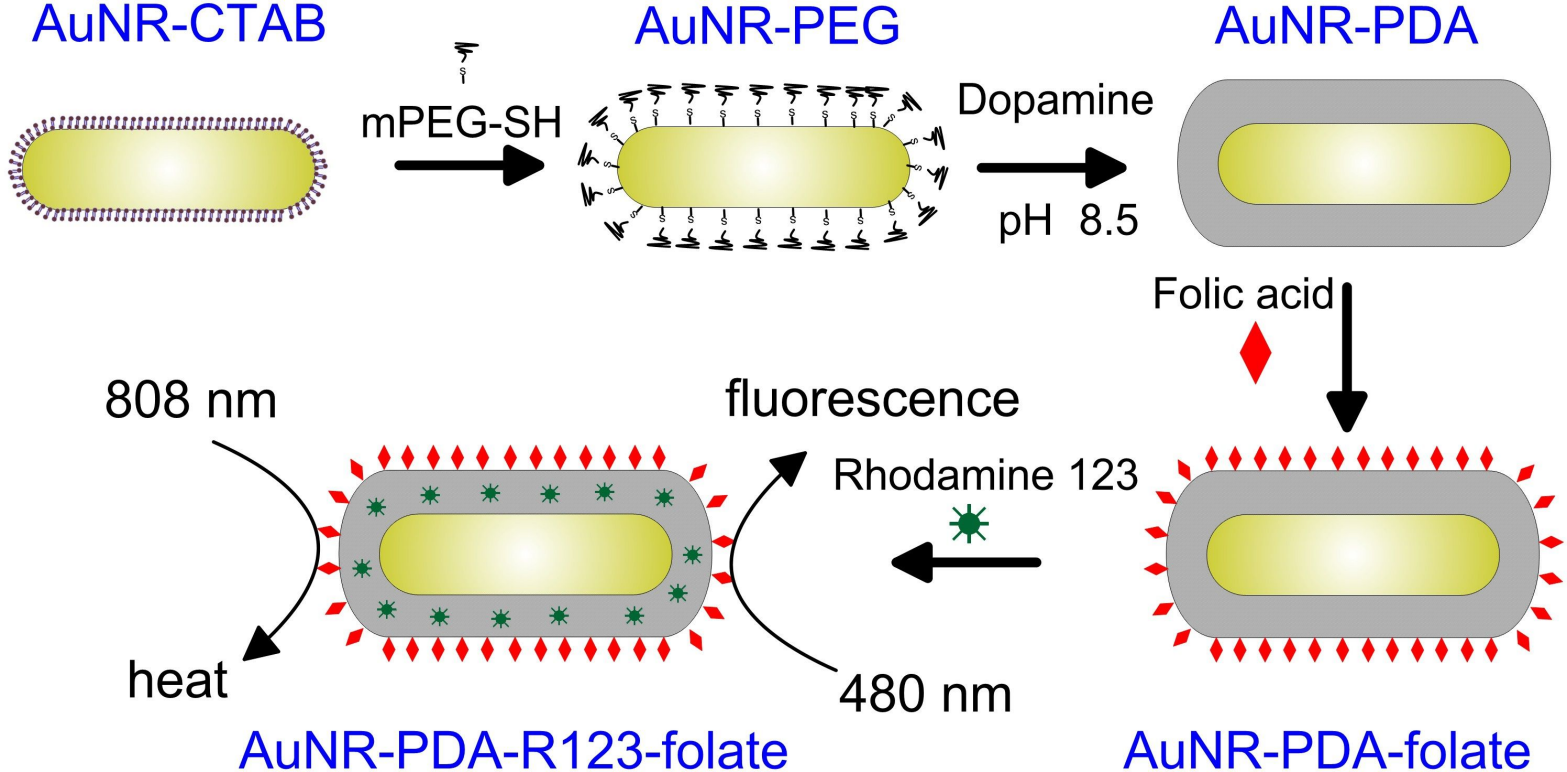
Schematic illustration of the synthesis, photothermal and fluorescence properties of the AuNR-PDA-R123-folate nanocomposites.

Herein, we present PDA-coated NIR-absorbing AuNRs and used the potential of the PDA layer for folate surface functionalization and rhodamine 123 (R123) loading, resulting in the formation of AuNR-PDA-R123-folate nanocomposites (Scheme 1).

This platform demonstrates three distinct features: (1) targeting of nanocomposites with folic acid leads to enhanced cellular uptake by folate-positive cancer cells compared with PEG-coated nanorods; (2) the high loading with rhodamine 123 makes the nanoparticles suitable for cell imaging with a simple fluorescent microscope; (3) through using NIR-mediated photothermal therapy the cancer cells can be killed with a high efficiency.

## Results and Discussion

### Synthesis and characterization of the AuNRs-PDA-R123-folate nanocomposite

**Figure 1:**
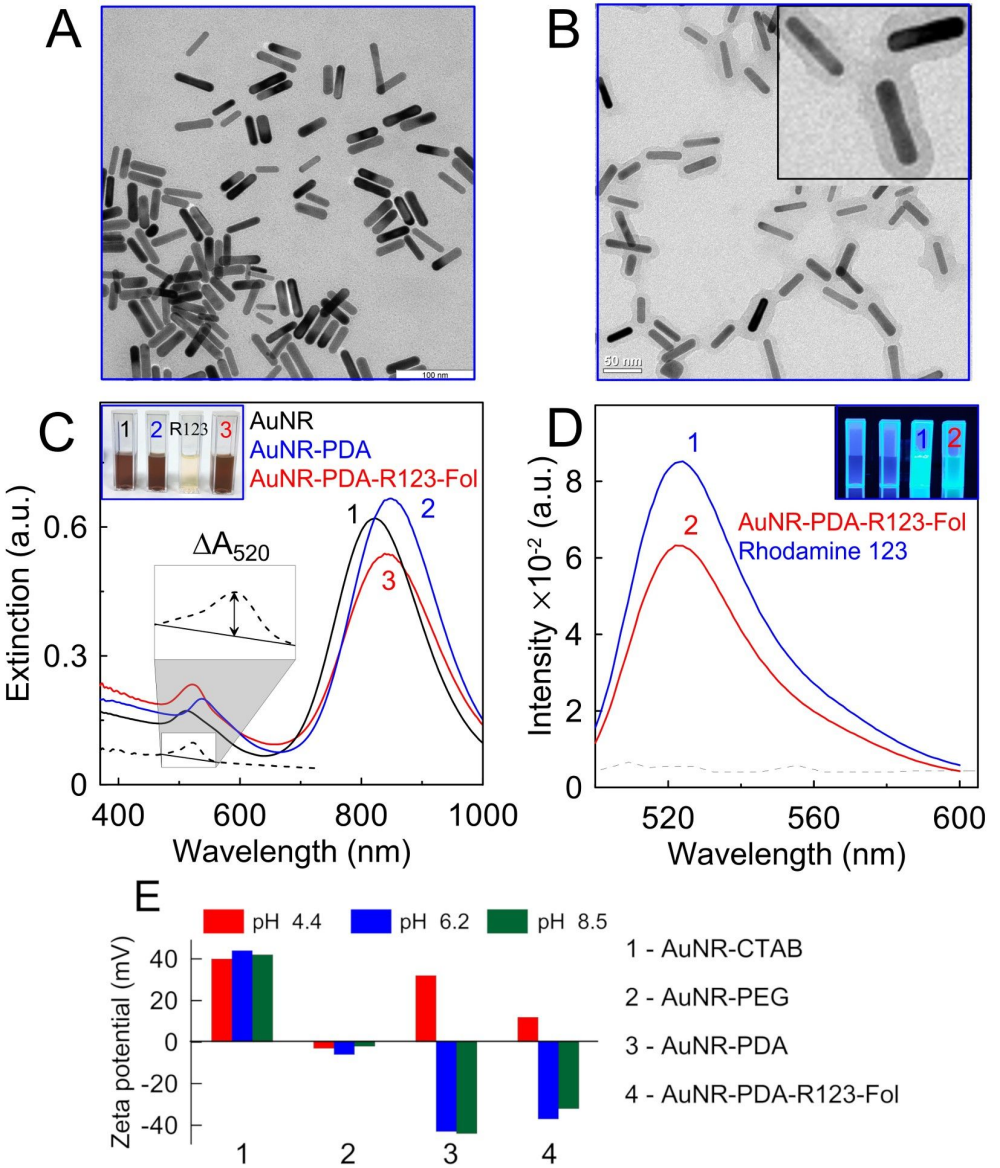
(A) Representative TEM image of AuNRs and (B) representative TEM image of PDA-coated AuNRs. The insert shows a magnified image. (C) Extinction spectra of as-prepared AuNRs (curve 1), PDA-coated AuNRs (curve 2), and AuNRs-PDA-R123-folate nanocomposite particles (curve 3). The dashed curve shows the differential spectrum of AuNR-PDA before and after loading of rhodamine 123. The insert shows a photo of cuvettes with as-prepared AuNRs, PDA-coated AuNRs, 1.5 µM rhodamine 123 solution and AuNRs-PDA-R123-folate nanocomposite particles under white-light illumination. (D) Fluorescence spectra of 1.5 µM rhodamine 123 solution (curve 1) and AuNRs-PDA-R123-folate nanocomposite particles (curve 2). The inset shows a photo of cuvettes with as-prepared AuNRs, PDA-coated AuNRs, 1.5 µM rhodamine 123 solution, and AuNRs-PDA-R123-folate nanocomposite particles under UV illumination. Pictures were taken with a UV Transilluminator Vilber Lourmat at 365 nm wavelength. (E) Zeta potential of AuNR-CTAB (1), AuNR-PEG (2), AuNR-PDA (3), and AuNR-PDA-R123-folate (4) nanocomposites measured at pH values of 4.4, 6.2, and 8.5.

AuNRs were fabricated by a seed-mediated method [41] with minor modifications concerning reaction protocol and some reagents, as described in [42–43]. According to statistical data derived from TEM images of 300 AuNRs (Figure 1A) the as-prepared particles have an average length of 44 ± 4 nm and an average width of 11 ± 2 nm.

These geometrical parameters lead to a longitudinal extinction peak at 810 nm (curve 1, Figure 1C). The transversal band located at 514 nm determines the orange-brown color of the suspension under white-light illumination (insert in Figure 1C). The ratio between transversal and longitudinal plasmonic peak intensities is 3.6, which is indicative to small amount of impurities in the AuNR sample.

The adsorption of positively charged CTAB molecules on AuNR surface prevents a successful adsorption of dopamine. To make a PDA coating feasible, the CTAB molecules were replaced with the thiolated polyethylene glycol (PEG-SH) as the latter molecules interact more strongly with the Au surface. Without this intermediate procedure, the adsorption of dopamine on CTAB-stabilized AuNRs resulted in nanoparticle aggregation. The formation of the PDA coating can be easily induced by polymerization of dopamine in alkaline environment. The polymerization process results in the formation of a PDA shell around the AuNR core (Scheme 1). The thickness of the PDA shell can be controlled by the concentration of dopamine added to reaction mixture.

In this study we mixed 10 mL of AuNRs with a concentration of 10^12^ mL^−1^ (a detailed calculation of nanorod concentration is given in Supporting Information File 1) with 1 mL of 3,4-dihydroxyphenethylamine (dopamine hydrochloride, DA) solution with a concentration of 1 mg/mL. As a result, nanorods were coated with a rough polymer shell having a thickness of 10 ± 3 nm. No uncoated AuNRs and free PDA particles were observed on the TEM images of the sample (Figure 1B). From an optical point of view the PDA coating leads to a red-shift of plasmon bands by 5–7 nm and sligth decrease in extinction. At the second stage, PDA-coated nanorods were functionalized with folates and rhodamine 123. Folate receptors are commonly overexpressed in cancer cells, e.g., in HeLa cells, enabling an easy targeting with folic acid [44]. Rhodamine 123 was used as a fluorescent dye to control the nanocomposite interaction with cells using fluorescent microscopy. Note, both folic acid and rhodamine 123 have amino groups and can be easily loaded to PDA by click conjugation [27]. In contrast, the loading efficiency for other well-known fluorescent FITC molecules into the PDA layer is decreased (data not shown). Figure 1C shows the extinction spectra of AuNRs, PDA-coated AuNRs and AuNRs-PDA-R123-folate nanocomposite particles. The inclusion of the rhodamine dye into the composite nanoparticles can be confirmed by the increased extinction in the wavelength region around 500 nm. The differential spectrum peak of AuNR-PDA composites before and after loading of rhodamine 123 corresponds to the absorption peak of rhodamine 123 in solution, thus confirming the successful inclusion of dye molecules into the PDA shell via amine groups through a simple one-step procedure.

The number of R123 molecules per composite particle can be estimated as follows. First, from the differential peak adsorption 0.035 and the calibration curve (Figure S2 in Supporting Information File 1), the molar concentration of bound dye molecules is 1.5 µM. Taking into account the estimated number concentration 10^12^ mL^−1^ of AuNRs (Section S1 in Supporting Information File 1), we obtain a loading efficiency of 10^3^ R123 molecules per one composite AuNRs-PDA-R123-folate particle. This number is comparable with the typical estimations of the loading capacity for AuNRs coated with a mesoporous silica shell [45] (several thousands of dye molecules per particle). In our case, a smaller loading capacity can be attributed to the relatively small AuNR size and to the difference in the chemical structure of the adsorbing molecules.

Thus, the PDA coating does not enhance the loading of both R123 and folate compared to the typical loading capacities reported for AuNRs coated with mesoporous silica shells. However, PDA coating makes the functionalization with any amine-containing ligand very simple and robust. It is sufficient to incubate PDA-coated nanorods with a desired functionalization component (for example, with folate and R123). Furthermore, the biocompatibility of the PDA biopolymer provides additional potential advantages for in vivo experiments as compared to other inorganic coatings such as silica shells.

It is well known [46] that the adsorption of fluorescent dyes on Au nanoparticles can induce quenching of their emission. However, in the AuNRs-PDA-R123-folate, the PDA layer prevents a direct contact between R123 molecules and AuNR. For fluorescent measurements the initial nanoparticle solution was diluted to 1/64th to prevent nonlinear behavior of the spectra related to the inner filter effect [47]. Figure 1D shows typical fluorescent emission spectra recorded at 480 nm excitation of free rhodamine 123 (curve 1) and AuNRs-PDA-R123-folate (curve 2) solutions at roughly equivalent concentrations of dye. The fluorescence intensity of R123 was found to be quenched with a quenching factor of about 0.75. As the rhodamine 123 molecules are well separated by a 10 nm PDA shell from the AuNR core, the energy transfer to the metal core seems to be low. Perhaps an additional quenching factor is due to the large surface density of the dye molecules loaded onto the composite particles. Straightforward evidence for a successful functionalization of the composite particles with R123 is depicted in the inset of the Figure 1D. Shown here are cuvettes containing as-prepared AuNRs, PDA-coated AuNRs, 1.5 µM rhodamine 123 solutions and AuNRs-PDA-R123-folate nanocomposite. When irradiated with a UV lamp, the latter two cuvettes exhibit intense blue-green fluorescent emission, whereas the first two cuvettes retain their dark color. It is notable that contrary to the usual physical loading of molecular content into mesoporous silica, the nanocomposites with PDA shell obtained by chemisorption remain stable without dye release during several washing steps and long-term storage.

For theranostic applications, the stability of nanocomposites under ambient conditions is a key factor. Here, we measured zeta potential and the particle-size distribution for all stages of nanocomposite synthesis. The measurements were made in citric buffer (pH 4.4), in water (pH 6.2), and Tris buffer (pH 8.5) (Figure 1E). The CTAB-coated AuNRs were positively charged (zeta potential of about +45 mV) independently of the pH value. The replacement of CTAB with PEG at the first synthetic stage resulted in an almost neutral particle charge (zeta potential varied from −2 to −4 mV). PDA-coated particles have a strong negative zeta potential of about −40 mV at neutral and alkaline pH values, whereas resuspension in acidic buffer leads to a recharging of particles, up to +35 mV. This process can be accompanied by particle aggregation when their charge is close to zero (see Figure S2 in Supporting Information File 1). Finally, the zeta potential of full nanocomposites AuNR-PDA-R123-folate demonstrated a dependence on the pH value similar to that of AuNR-PDA particles. The only difference was that the final nanocomposites demonstrated better colloidal stability and smaller absolute values of positive and negative zeta potentials under acidic and alkaline conditions, respectively.

For CTAB- and PEG-coated AuNRs, DLS measurements showed similar bimodal size distributions, corresponding to rotational and translational diffusion (Figure S2A,B, Supporting Information File 1). PDA-coated nanorods in Tris buffer (pH 8.5) demonstrated an increase in translation diffusion size and a decreasing contribution to the rotational diffusion due to the polymeric PDA shell (Figure S2C). With a decrease in pH value from 6.2 to 4.4 an evident aggregation tendency was observed from DLS size-distributions (Figure S2D,E). For complete nanocomposites, AuNR-PDA-R123-folate, the DLS size-distribution at pH 8.5 was virtually the same as for AuNR-PDA particles (Figure S2F). What is more, with a decrease in pH value, the final nanocomposites demonstrated better stability and small variations in DLS size distributions (Figure S2G,H).

Thus, we obtained composite nanoparticles that have two important theranostic modalities. First, due to the strong light absorption by the AuNR core, the nanoparticles are suitable for photothermal treatment in the NIR tissue transparency window. Second, the nanocomposite shows strong fluorescence under visible-light illumination due to the presence of R123 molecules. Additionally, nanoparticles can selectively accumulate in the cancer cells because of targeting to folate receptors.

### Folate-mediated cell imaging

**Figure 2:**
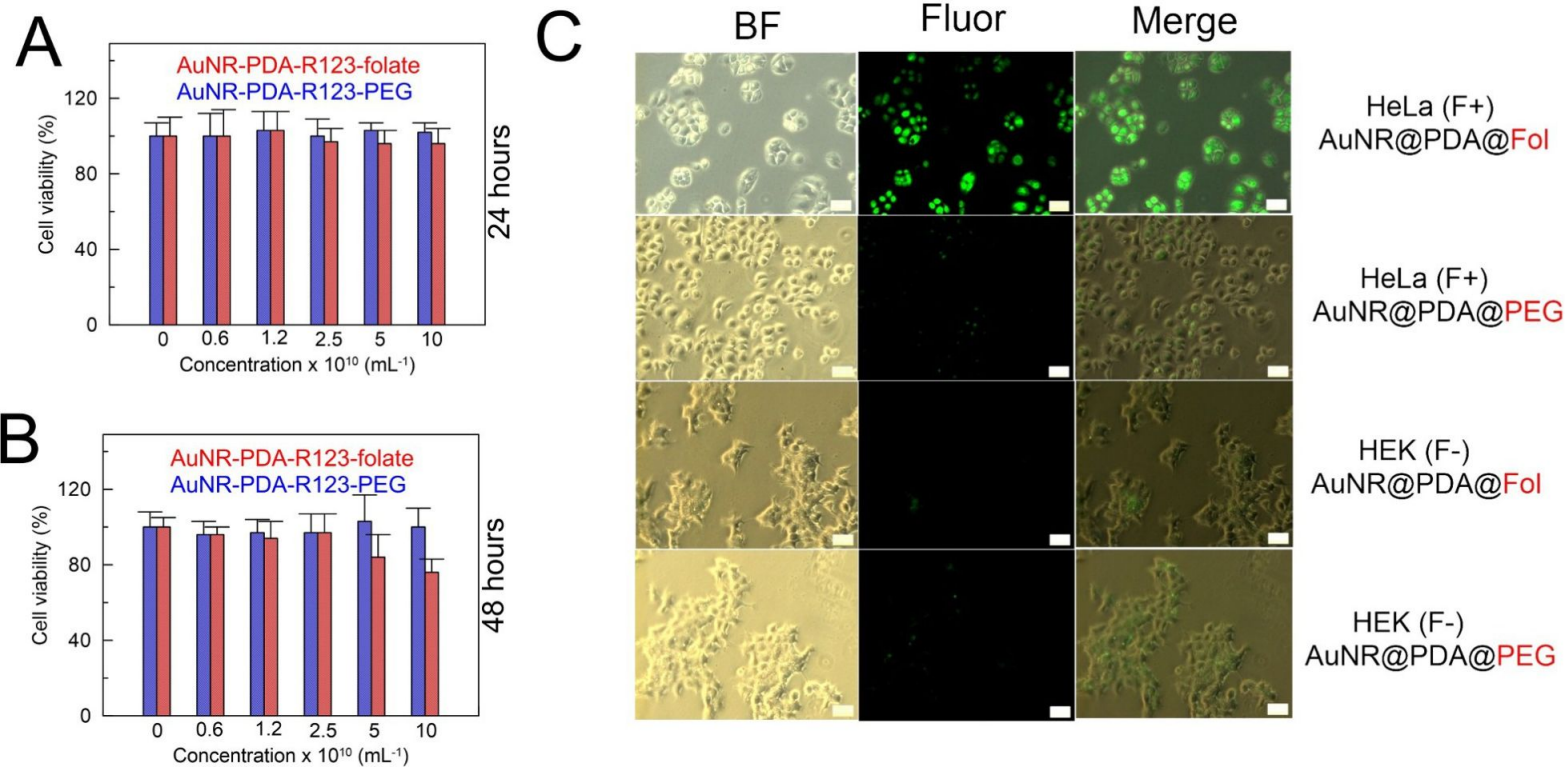
In vitro cell viability of HeLa cells incubated with various concentrations of AuNR-PDA-R123-folate or AuNR-PDA-R123-PEG after (A) 24 h and (B) 48 h of incubation. (C) The microscopy images of HeLa (F+) and HEK 293 (F−) cells after treatment with AuNR-PDA-R123-folate or AuNR-PDA-R123-PEG for 2 h. BF: bright-field imaging; Fluor: fluorescence imaging.

Efficient cellular uptake of nanocarriers is significant to ensure the therapeutic efficacy of plasmonic photothermal therapy. In this work we utilized fluorescent properties of our nanocomposites to study the folate-mediated nanoparticle uptake. Folate-positive HeLa and folate-negative HEK 293 cell lines were used as models. PEGylated AuNRs-PDA-R123-PEG particles were used as a reference to estimate nonspecific uptake. To understand the safe dose of PEG-coated and folate-functionalized polydopamine-encapsulated AuNRs, we investigated their biocompatibility by a standard resazurin-based cytotoxic assay. Both folate-functionalized and PEG-coated nanocomposites incubated with HeLa cells during 24 and 48 h demonstrated insignificant cell toxicity (Figure 2A,B) for particle concentrations up to 2.5 × 10^10^ mL^−1^. At very high concentrations and after 48 h of incubation time the cell viability decreased to 83% and 76% only for AuNRs-PDA-R123-folate. We attributed this effect to a better uptake of targeted nanoparticles compared with PEG-coated particles.

HeLa (F+) and HEK 293 (F−) cells were incubated with AuNR-PDA-R123-folate and AuNR-PDA-R123-PEG at a nanoparticle dose of 10^10^ mL^−1^ for 2 h. Fluorescent images of randomly selected cells under 488 nm light excitation were obtained using a Leica DM 2500 fluorescent microscope.

As shown in Figure 2C, the fluorescence signal inside HeLa cells could be detected after the incubation of cells with folate-targeted nanocomposites, illustrating that AuNR-PDA-R123-folate could be effectively internalized into HeLa cells. The nonspecific uptake of PEG-coated nanocomposites by HeLa cells is relatively low. The folate-negative HEK 293 cells demonstrate equally low uptake of folate- and PEG-functionalized nanocomposites. These results show that the folic acid targeted nanoprobe can be used to detect folate-positive cancer cells through fluorescent imaging.

### In vitro photothermal effects

**Figure 3:**
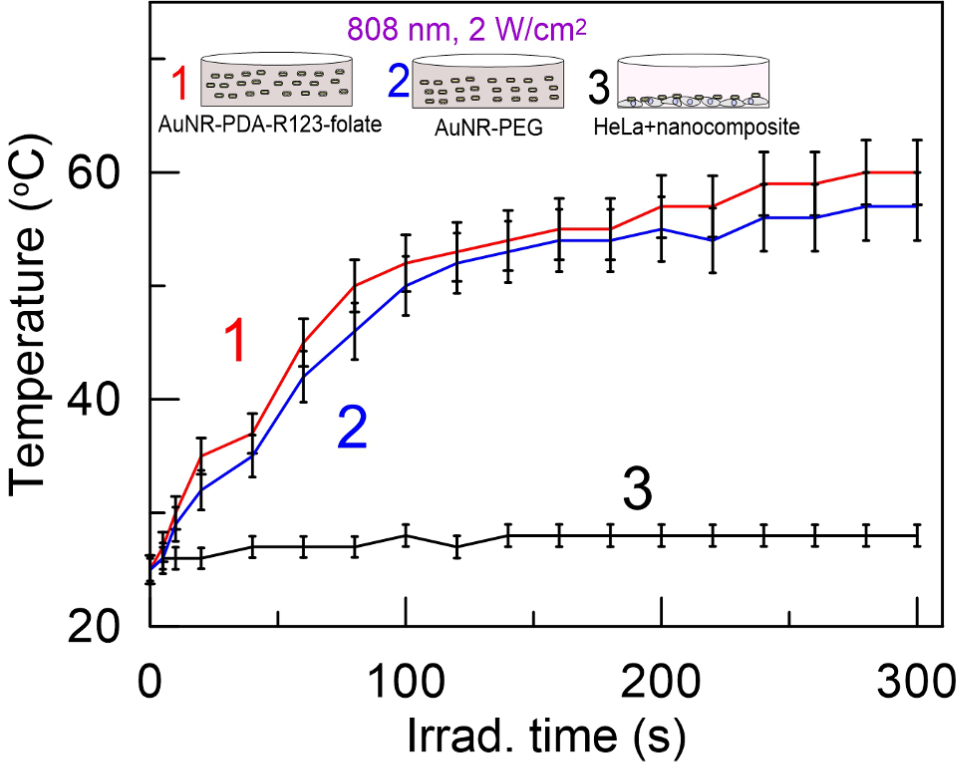
Temperature changes of AuNR-PDA-R123-folate (curve 1), AuNR-PEG (curve 2) solutions, and well with adhered HeLa cells (curve 3) incubated with AuNR-PDA-R123-folate in response to irradiation of a NIR laser (808 nm) with a power density of 2 W/cm^2^.

Notably, the AuNR-PDA-R123-folate not only served as fluorescent imaging agent, but it was also employed as a NIR light absorber in photothermal laser therapy. To compare the photothermal properties of PDA-coated nanorods with PEG-coated nanorods, we added 200 µL of AuNR-PDA-R123-folate or AuNR-PEG in standard 96-well plates and subsequently illuminated with 808 nm CW laser at 2 W/cm^2^ for 300 s. The concentration of the nanoparticles was 10^11^ mL^−1^. The temperature in the wells was directly measured every 20 s during the irradiation by employing a contact microthermometer. Curve 1 and curve 2 in Figure 3 show that the temperature of the well solutions increased more rapidly during the first 100 s of the irradiation and reached 60 °C after 300 s of irradiation. Importantly, no significant difference in photothermal response of PEG- and PDA-coated AuNRs was observed.

A temperature of 60 °C in the well is sufficient to obtain complete cell death after a dozen of seconds [48]. On the other hand, the efficiency of photothermal treatment is usually connected to a local increase in temperature around cancer cells or tissues without heating of the solution or surrounding tissues. Curve 3 in Figure 3 shows small temperature changes in the well with grown HeLa cells incubated with AuNR-PDA-R123-folate nanocomposites in response to the laser irradiation. In this experiment, the HeLa cells were incubated with nanoparticles (concentration 10^11^ mL^−1^), added to the cell medium for 2 h, than the excess nanoparticles were replaced by fresh non-supplemented DMEM medium before irradiation. It should be emphasized that the total number of bound nanorods in sample 3 is significantly lower compared to the number of free nanorods in samples 1 and 2. As a result, we observed no increase in the bulk temperature of the solution of sample 3 measured directly in the wells. Thus, a possible influence of NIR irradiation on the cell viability can be attributed to the local heating effect [49] rather than to the total increase of the solution temperature. Depending on the experimental conditions (nanoparticle adsorption and uptake, the irradiation fluence, CW or pulsed irradiation regime), the local heating is not harmful to the treated cells and can be used as a physical way for laser optoporation and controlled release [50]. In our case, the localized character of heating at 2 W/cm^2^ of NIR CW irradiation corresponded to a typical hyperthermia of cells [51–52].

**Figure 4:**
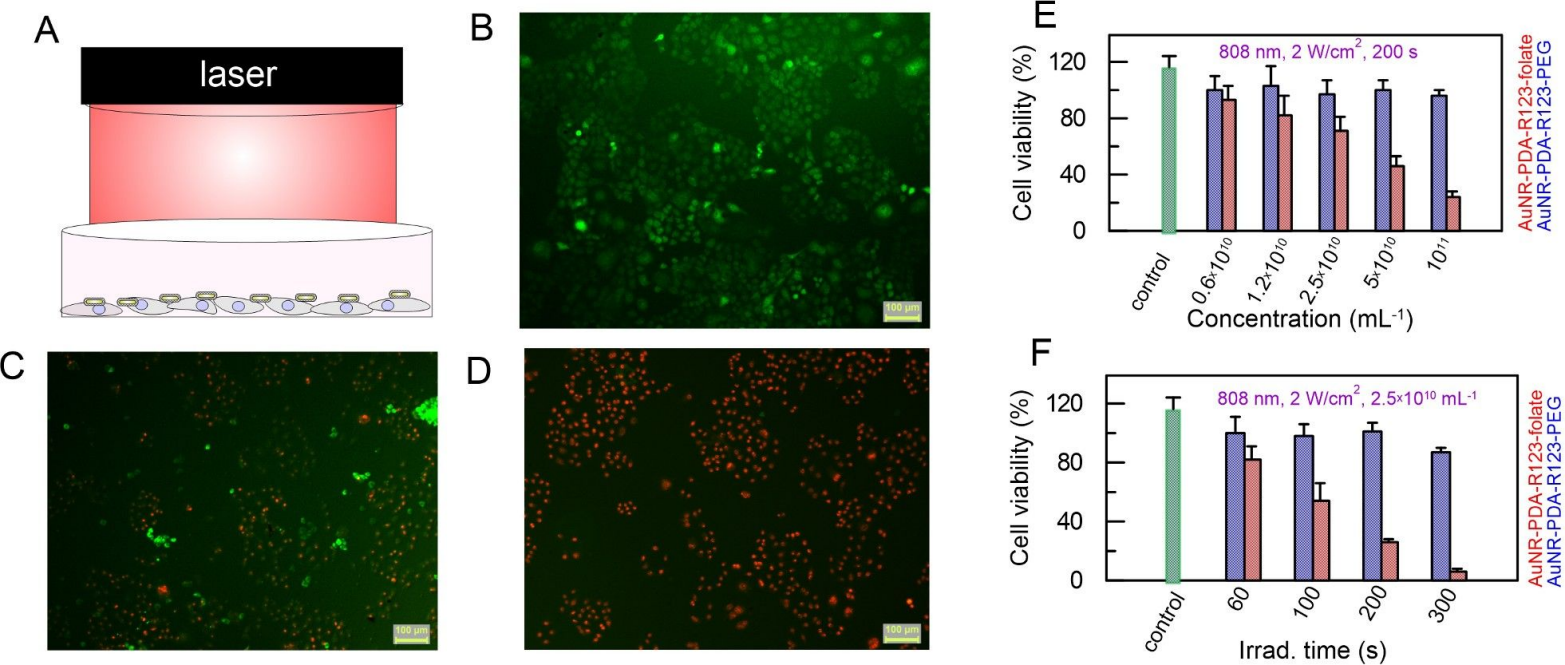
Photothermal therapy of Hela cells in vitro. (A) Scheme of Hela cells irradiation; (B) fluorescence images of FDA/PI co-stained (live cells are green, dead cells are red) samples after targeted photothermal ablation: control, non-irradiated cells; cells incubated with 5 × 10^10^ mL^−1^ AuNR-PDA-R123-folate after (C) 60 s and (D) 300 s of 808 nm irradiation at 2 W/cm^2^. The scale bar is 100 μm. (E) Cell viabilities estimated through standard resazurin assay after photothermal ablation using AuNR-PDA-R123-folate (red bars) and AuNR-PDA-R123-PEG (blue bars) at different concentrations, other parameters were 808 nm laser, 2 W/cm^2^, 200 s. (F) Cell viabilities after photothermal ablation using AuNR-PDA-R123-folate (red bars) and AuNR-PDA-R123-PEG (blue bars) at different irradiation times, other parameters were 808 nm laser, 2 W/cm^2^, nanoparticles concentration 2.5 × 10^10^ mL^−1^.

To investigate the role of folate targeting in photothermal cancer therapy, folate-positive HeLa cells were incubated with AuNR-PDA-R123-folate or AuNR-PDA-R123-PEG nanocomposites of different concentrations. After incubation, the cultural medium containing unbound nanoparticles was removed and fresh DMEM was added to each well. The cells were irradiated with 808 nm laser light to initiate photothermal ablation (Figure 4A). The laser-treated cells were further stained with fluorescein diacetate and propidium iodide (FDA/PI) dyes, coloring live cells in green and apoptotic cells in red (Figure 4B–D).

To quantify the efficiency of treatment the cell viability was estimated by using the resazurin assay. After irradiation with the NIR laser for 200 s, HeLa cells treated with AuNR-PDA-R123-folate exhibited a dose-dependent cell viability (Figure 4E, red columns) from 90% for the lowest concentration to 30% for the highest concentration. In comparison, the cell viability after treatment with PEG-coated nanoparticles is well preserved under the same experimental conditions (Figure 4E, blue columns).

At a fixed nanoparticle concentration and a laser power density of 2 W/cm^2^, the in vitro photothermal ablation was further studied as a function of the irradiation time (Figure 4F). As the irradiation time was increased from 60 to 300 s, the relative cell viability gradually decreased for both nanoparticles samples (Figure 1E). Obviously, the effect of folate-targeted photothermal ablation is higher. For example, at a concentration of 2.5 × 10^10^ mL^−1^, a power density of 2 W/cm^2^, and an irradiation time of 300 s, the cell viability was lower than 10% when the folate-targeted nanocomposite was applied. In comparison, the cell viability remains at around 80% for the cells treated with PEG-coated nanoparticles.

## Conclusion

In this work, nanocomposites with an AuNR core, and a polydopamine shell loaded with fluorescent dye (rhodamine 123) and functionalized with folic acid have been successfully fabricated. These novel nanocomposites have uniform size distributions, are stable in aqueous solution, nontoxic, demonstrate plasmonic extinction under NIR excitation, strong fluorescence under UV–vis excitation and the ability to selectively accumulate in folate-positive cancer cells. By using fluorescent microscopy we demonstrated that folate-functionalized nanoparticles can selectively accumulate in folate-positive HeLa but not in folate-negative HEK 293. The targeted NIR light-mediated phototherapy by using AuNR-PDA-folate showed remarkable cancer cell killing efficiency in vitro in comparison with AuNR-PEG nanoparticles.

The feature renders the nanocomposites very attractive due to their ability to implement folate-mediated fluorescent imaging and photothermal ablation of cancer cells.

## Experimental

### Reagents

Dopamine hydrochloride (DA, H8502), cetyltrimethylammonium bromide (CTAB, >98.0%), cetyltrimethylammonium chloride (CTAC, 25% water solution), L-ascorbic acid (AA, >99.9%), hydrochloric acid (HCl, 37 wt % in water), folic acid (99.9%), thiolated polyethylene glycol (mPEG-SH, 99%), rhodamine 123 (BioReagent, for fluorescence, ≥85%) and sodium borohydride (NaBH_4_, 99%) were purchased from Sigma-Aldrich. Hydrogen tetrachloroaurate trihydrate (HAuCl_4_·3H_2_O) and silver nitrate (AgNO_3_, >99%) were purchased from Alfa Aesar. Ultrapure water obtained from a Milli-Q Integral 5 system was used in all experiments.

### Synthesis of AuNRs

AuNRs with a plasmon peak at around 800 nm were obtained by the seed-mediated growth method [41]. First, gold seed particles were prepared by adding 0.1 mL of sodium borohydride (10 mM) to 10 mL of 0.25 mM HAuCl_4_ in 100 mM CTAB. Next, 1 mL of 4 mM AgNO_3_, 2.5 mL of 10 M HAuCl_4_, 0.5 mL of 80 mM isoascorbic acid, 0.5 mL of 1 М HCl, and 0.5 mL of gold seed solution are sequentially added to 50 mL of 0.1 M CTAB solution. AuNRs were allowed to grow overnight without stirring at 30 °C. For further PDA coatings the prepared nanorods were PEGylated using the procedure described in [53].

### Polydopamine coating of AuNRs

PDA shells were grown on the surface of PEGylated nanorods. To this end AuNRs were centrifuged (12000*g*, 30 min) and resuspended in 10 mM Tris buffer (pH 8.5). A dopamine (DA) solution with an initial concentration of 1 mg/mL was freshly prepared in water. Next, 1 mL of DA solution was quickly injected into 10 mL of AuNRs suspension under sonication and allowed to react for 3 h at room temperature. The as-synthesized PDA-coated nanorods were purified by repeated centrifugation at 12000*g* for 15 min and finally resuspended in 10 mL of PBS buffer (pH 7.4).

### Functionalization with folate and loading with rhodamine 123

10 mg of folic acid was dissolved in 1 mL DMSO. To immobilize the folate, 10 µL of folic acid solution in DMSO was added to 10 mL AuNR-PDA suspension, sonicated for 5 min and kept undisturbed for 24 h. To remove unbound components, the suspension was then centrifuged at 12000*g* for 10 min, the supernatant was decanted and the pellet was resuspended in 10 mM PBS buffer.

### AuNR-PDA-folate nanoparticles

For a comparative study, PEG-coated AuNR-PDA was used instead of folate-conjugated nanorods. To this end, 100 µL of 1 mM PEG-SH was added to 10 mL AuNR-PDA suspensions. To remove unbound components, the suspension was then centrifuged at 12000*g* for 10 min, the supernatant was decanted and the pellet was resuspended in 10 mM PBS buffer. The resulted nanoparticles were denominated AuNR-PDA-PEG.

Next, 100 µL of rhodamine 123 solution in DMSO (20 µM) was mixed with AuNR-PDA-folate at 25 °C for 2 h, yielding the AuNR-PDA-R123-folate nanocomposite. To remove unbound components, the suspension was then centrifuged at 12000*g* for 10 min twice, the supernatant was decanted and the pellet was resuspended in 10 mM PBS buffer or serum-free DMEM.

### Measurements

Extinction spectra were measured with a Specord 250 spectrophotometer (Analytik, Jena, Germany). Transmission electron microscopy (TEM) images were recorded with a Libra-120 transmission electron microscope (Carl Zeiss, Jena, Germany) at the Simbioz Center for the Collective Use of Research Equipment in the Field of Physical–Chemical Biology and Nanobiotechnology, IBPPM RAS, Saratov. For measurements of visible fluorescence spectra, we used a Cary Eclipse spectrofluorometer.

### Cytotoxicity assay

The in vitro cytotoxicity was measured using a standard resazurin (Alamar blue) assay following the manufacturer instructions. HeLa cells (1 × 10^5^ cells/well) were seeded into 96-well cell-culture plate and then incubated for 24 h at 37 °C under 5% CO_2_. Then the different concentrations (from 0.6 × 10^10^ to 10^11^ mL^−1^) of AuNRs-PDA-R123-folate and AuNRs-PDA-R123-PEG, dispersed in DMEM, were added and incubated for another 24 h or 48 h. 1 mM resazurin sodium salt in PBS (10 µL) was added directly to each well, the plates were incubated at 37 °C to allow cells to convert resazurin to resorufin, and the fluorescence signal was measured at the 600 nm wavelength using a Cary Eclipse spectrofluorimeter equipped with a plate reader.

### Targeted cellular imaging

Folate-positive HeLa and folate-negative HEK 293 cells were seeded on the surface of 24 mm microscopic glass coverslips at a density of 10^5^ cells per well for 24 h and allowed to grow to ca. 50% confluence. The culture medium was then replaced with serum-free DMEM containing AuNRs-PDA-R123-folate and AuNRs-PDA-R123-PEG nanoparticles (concentration 100 pM) for 2 h. After incubation, the cells were washed three times with PBS to remove the excess nanoparticles. Fluorescent and transmitted light microscopy images were obtained with a Leica 2500 DM microscope using phase contrast (white light) and FL (excitation at 480 nm) modes.

### Plasmonic photothermal therapy in vitro

To evaluate the thermal therapeutic effect of the probe, the HeLa cells were incubated with AuNRs-PDA-R123-folate and AuNRs-PDA-R123-PEG at the given concentrations for 2 h. After incubation, the cells were washed twice with PBS (pH 7.4) and supplemented with fresh culture medium. Subsequently, the cells were treated with a NIR laser (808 nm, 2 W/cm^2^) for 60–300 s. 2 h after the procedure the treated cells were stained with a dye mixture, FDA (2 μM) and PI (4 μM), to indicate the live and dead cells, and examined by FL microscopy, successively. The cell viability after NIR laser exposure was also examined using the resazurin assay.

## Supporting Information

S1. Calculation of the AuNR concentration; S2. Calibration curve for determination of rhodamine 123 concentration; S3. Dynamic light scattering study of nanocomposites at different pH values.

File 1 Additional experimental data.
