# Supplementary material for: Polydopamine-coated Au nanorods for targeted fluorescent cell imaging and photothermal therapy
Source: Beilstein J Nanotechnol. 2019 Apr 1;10:794–803. doi: 10.3762/bjnano.10.79 (PMC6466791; doi:10.3762/bjnano.10.79)
Supplement: File 1 — Additional experimental data. [file Beilstein_J_Nanotechnol-10-794-s001.pdf]

BEILSTEIN JOURNAL OF NANOTECHNOLOGY

Supporting Information

for

Polydopamine-coated Au nanorods for targeted fluorescent cell imaging and photothermal therapy

Boris N. Khlebtsov, Andrey M. Burov, Timofey E. Pylaev and Nikolai G. Khlebtsov

Beilstein J. Nanotechnol. **2019,** *10,* 794–803. doi:10.3762/bjnano.10.79

Additional experimental data

License and Terms: This is a supporting information file under the terms of the Creative Commons Attribution License (http://creativecommons.org/licenses/by/4.0). Please note that the reuse, redistribution and reproduction in particular requires that the authors and source are credited.
The license is subject to the *Beilstein Journal of Nanotechnology* terms and conditions: (https://www.beilstein-journals.org/bjnano)

S1. Calculation of the AuNR concentration

According our synthetic procedure, the final Au concentration is 0.5 mM. Let assume yield of nanorods in reaction about 85%, thus mass/volume concentration of Au is about $c \approx 85$ μg/mL. According TEM data, AuNRs have s-cylinder shape with average length of L = 44 nm and width (diameter) of d = 11 nm. The volume of a single nanorod is $V_1 = \frac{1}{6}\pi d^3 + \pi(L-d)\frac{d^2}{4} = 697 + 3136 = 3833$ nm^3. With gold density $\rho = 19.32$ g/cm^3, the average mass of a single nanorod is $m_1 = \rho V_1 = 7.64 \times 10^{-11}$ g. The number concentration of nanorods is $N = c / m_1 = 1.12 \times 10^{12}$ mL^{-1}. Taking into account all approximation made, we accume the final number concentration to be about 10^{12} particles per mL.

S2. Calibration curve for determination of rhodamine 123 concentration

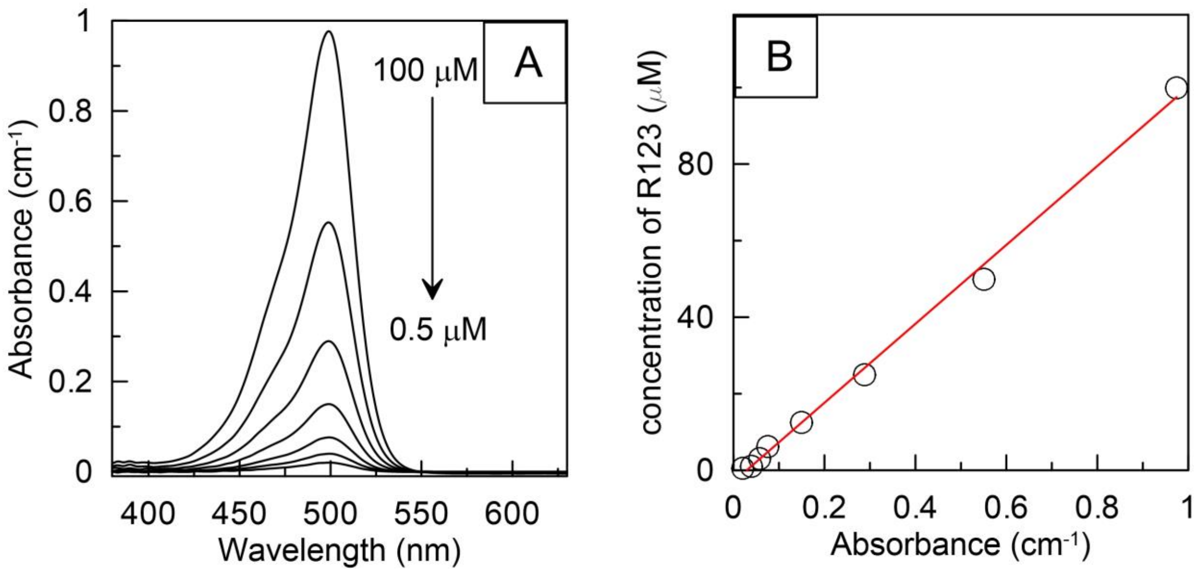

Figure S1: (A) Absorbance spectra of rhodamine 123 solutions with concentration from 100 to 0.5 μM (double dilutions) measured in 1 cm cuvette. (B) Calibration curve for determination of rhodamine 123 concentration from extinction measurements. Absorbance is measured at peak wavelength 500 nm.

S3. Dynamic light scattering study of nanocomposites at various pH

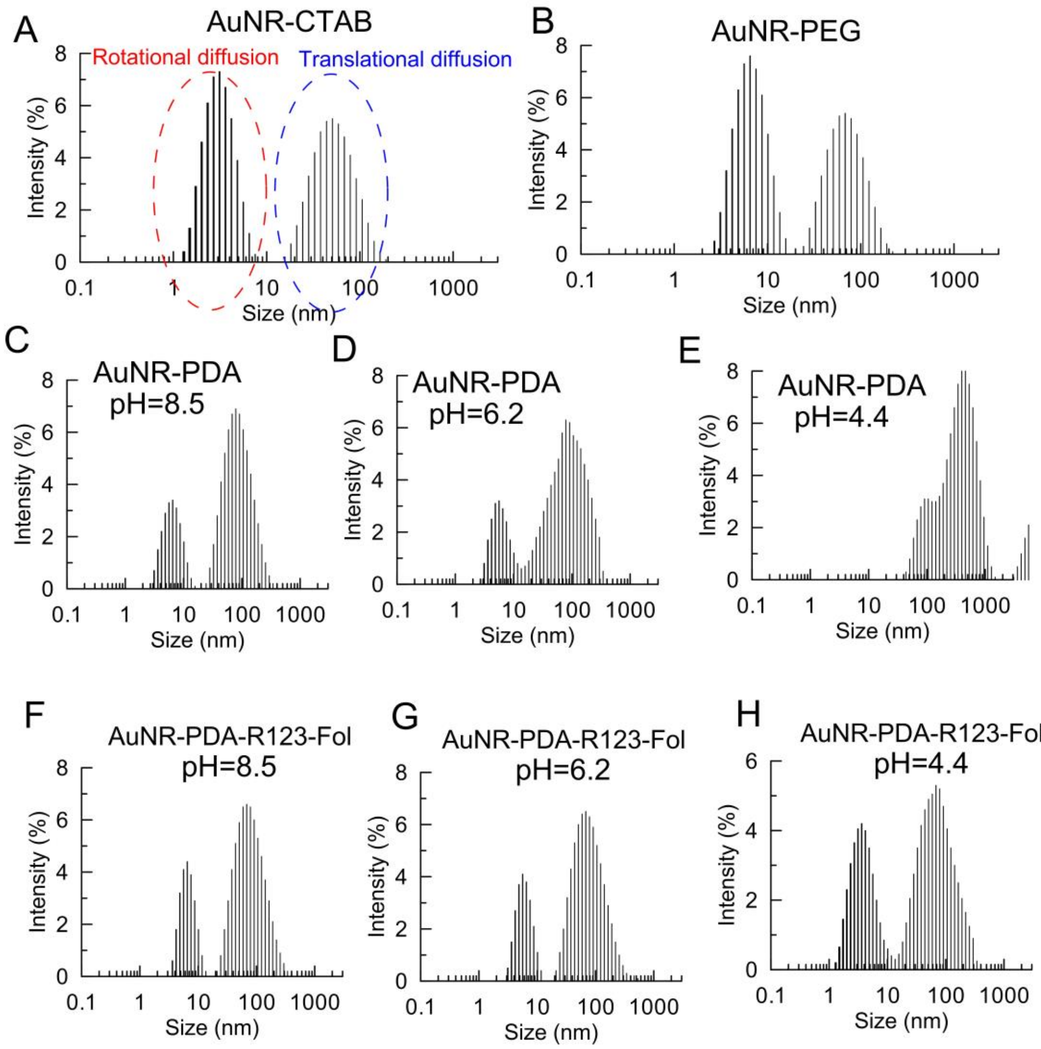

Figure S2: DLS size-distributions of AuNR-CTAB (A) and AuNR-PEG (B) in water; AuNR-PDA (C–E) and AuNR-PDA-R123-Folate (F–H) measured at pH 8.5 (C,F), 6.2 (D,G), and 4.4 (E,H).
